# Licochalcone D from *Glycyrrhiza uralensis* Improves High-Glucose-Induced Insulin Resistance in Hepatocytes

**DOI:** 10.3390/ijms251810066

**Published:** 2024-09-19

**Authors:** Yu Geon Lee, Hee Min Lee, Jin-Taek Hwang, Hyo-Kyoung Choi

**Affiliations:** 1 Personalized Diet Research Group, Korea Food Research Institute (KFRI), Wanju 55365, Republic of Korea; ugun2@kfri.re.kr (Y.G.L.); jthwang@kfri.re.kr (J.-T.H.); 2 Kimchi Industry Promotion Division, World Institute of Kimchi, Gwangju 61755, Republic of Korea; hmlee@wikim.re.kr

**Keywords:** licochalcone D, *Glycyrrhiza uralensis*, insulin resistance, glucose metabolism, hepatocytes

## Abstract

This study investigated the therapeutic potential of licochalcone D (LicoD), which is derived from *Glycyrrhiza uralensis*, for improving glucose metabolism in AML12 hepatocytes with high-glucose-induced insulin resistance (IR). Ultra-high-performance liquid chromatography–mass spectrometry revealed that the LicoD content of *G. uralensis* was 8.61 µg/100 mg in the ethanol extract (GUE) and 0.85 µg/100 mg in the hot water extract. GUE and LicoD enhanced glucose consumption and uptake, as well as *Glut2* mRNA expression, in high-glucose-induced IR AML12 cells. These effects were associated with the activation of the insulin receptor substrate/phosphatidylinositol-3 kinase signaling pathway, increased protein kinase B α phosphorylation, and suppression of gluconeogenesis-related genes, such as *Pepck* and *G6pase*. Furthermore, GUE and LicoD promoted glycogen synthesis by downregulating glycogen phosphorylase. Furthermore, LicoD and GUE mitigated the downregulated expression of mitochondrial oxidative phosphorylation proteins in IR hepatocytes by activating the PPARα/PGC1α pathway and increasing the mitochondrial DNA content. These findings demonstrate the potential of LicoD and GUE as therapeutic options for alleviating IR-induced metabolic disorders by improving glucose metabolism and mitochondrial function.

## 1. Introduction

Insulin resistance (IR) is a serious condition that causes metabolic abnormalities that disrupt the balance between energy consumption and expenditure [1]. IR impairs the ability of muscle, fat, and liver cells to absorb glucose from the bloodstream in response to insulin, resulting in elevated blood glucose levels (hyperglycemia) [2]. Impaired glucose metabolism can trigger an inflammatory response that leads to the development of type 2 diabetes (T2DM), nonalcoholic fatty liver disease (NAFLD), and obesity [3]. Consequently, significant research has focused on identifying pharmaceutical options that can improve IR to prevent or treat various metabolic diseases [4].

Glucose metabolism in hepatocytes requires the activation of insulin receptor substrates (IRSs) and the phosphatidylinositol-3 kinase a(PI3K) signaling pathway [5]. This pathway plays a significant role in regulating the expression of metabolic genes involved in both glucose production and storage [5]. Furthermore, because the IRS/PI3K signaling pathway ensures that hepatocytes respond effectively to insulin to maintain glucose homeostasis, its disruption is closely associated with IR-induced metabolic disorders [6]. Therefore, improving insulin-mediated glucose metabolism by targeting the IRS/PI3K signaling pathway is an effective strategy for alleviating metabolic diseases, including T2DM [7].

The regulation of net glycogen synthesis is a critical physiological function of insulin signaling in hepatocytes. IR-induced hyperglycemia not only inhibits hepatic glycogen synthesis but also activates glycogen phosphorylase, the enzyme responsible for metabolizing glycogen into glucose [8]. This process induces muscle mass loss. One important function of protein kinase B α (Akt), which is activated by the IRS/PI3K signaling pathway, is to inactivate glycogen phosphorylase, thereby inhibiting glycogen breakdown [9]. Furthermore, insulin signaling through the IRS/PI3K/Akt pathway activates the expression of genes related to glycogen synthesis, including the regulation of glycogen synthase kinase 3 (GSK3) [10]. Decreased transcriptional repression of GSK3 occurs in patients with IR, indicating that GSK3 remains highly active, inhibiting glycogen synthase and further disrupting glycogen synthesis [11]. Therefore, targeted regulation of the activities of glycogen phosphorylase and glycogen synthase effectively prevent and improve IR-induced metabolic disorders.

Mitochondria are essential organelles for glucose metabolism that generate cellular energy via oxidative phosphorylation (OXPHOS) [12]. The interplay between mitochondrial function and metabolic disorders has gained significant attention, highlighting the crucial role of mitochondrial dysfunction in the progression of metabolic diseases, including T2DM [13]. In particular, IR in hepatocytes, which results in abnormal glucose disposal, is closely associated with mitochondrial dysfunction [14]. Thus, impaired glucose consumption in the IR state contributes to diminished mitochondrial energy production, thereby promoting the development of metabolic disorders [15]. Therefore, strategies targeting mitochondrial functions that enhance oxidative capacity or protein quality could help prevent and treat IR-induced metabolic diseases [16].

**Figure 1:**
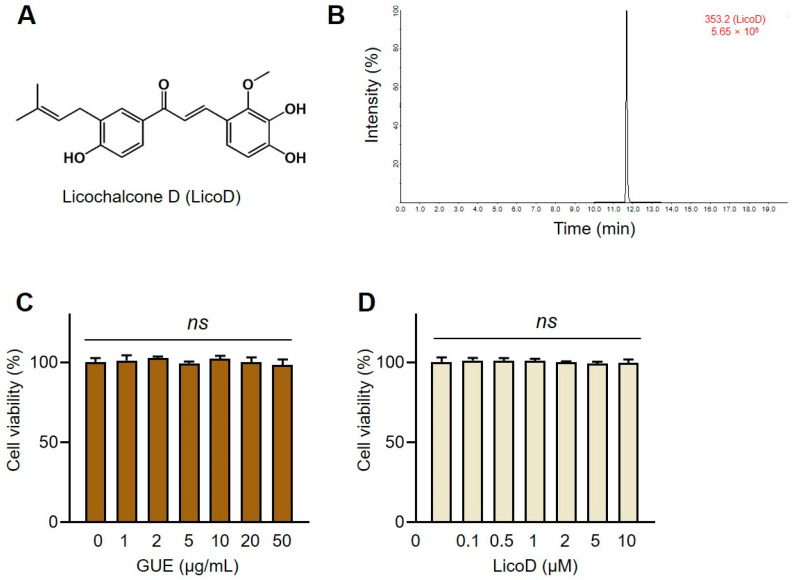
Quantitative analysis of licochalcone D (LicoD) from *Glycyrrhiza uralensis* ethanol extract (GUE) and their cytotoxicity to hepatocytes. (**A**) Chemical structure of LicoD. (**B**) Ultra-high-performance liquid chromatogram of LicoD and GUE; multiple reaction monitoring transition *m*/*z* 353.2 [M–H]^−^ 150.0 [M–H]^−^. (**C**) The cell viability under (**C**) GUE or (**D**) LicoD treatment was measured using a water-soluble tetrazolium salt-1 kit. Data are expressed as mean ± standard deviation (*n* ≥ 3). The different letters indicate significant differences (*p* < 0.05) as determined by one-way analysis of variance (ANOVA) followed by Tukey’s post hoc test. ns, not significant.

*Glycyrrhiza* species (approximately 30 species), commonly known as licorice, are widely distributed in Eastern and Western countries [17]. Among them, *Glycyrrhiza uralensis* (*G. uralensis*) accounts for over 90% of licorice production used in traditional medicine due to its numerous health benefits, including anti-oxidative, anti-inflammatory, and anti-cancer properties [18,19]. *G. uralensis* is particularly renowned for its rich content of flavonoids, especially licochalcones, which contribute to many of its potent pharmacological effects [20]. Licochalcone D (LicoD, Figure 1A) is a significant chalcone that exerts antitumor effects by regulating multiple signaling pathways [21]. However, few studies have focused on quantifying LicoD in licorice and investigating its effects on IR-induced metabolic disorders.

This study quantified the LicoD content of *G. uralensis* ethanol extract (GUE) and hot water extract (GUH) and explored the effects of LicoD on glucose metabolism in high-glucose-induced IR hepatocytes.

## 2. Results

### 2.1. Determination of LicoD Contents in G. uralensis

We conducted UPLC–MS analysis to quantify the LicoD content in both GUE and GUH. LicoD had a retention time of 11.6 min on the total ion chromatogram (Figure 1B). The LicoD content was 8.61 ± 0.51 µg in GUE and 0.85 ± 0.06 µg in GUH, both expressed per 100 mg dry weight of *G. uralensis* roots (Table 1). Further experiments were performed using GUE to investigate whether *G. uralensis* and its active component, LicoD, could modulate glucose metabolism by alleviating IR-induced metabolic disorders. We also assessed the cytotoxicity of GUE and LicoD on hepatocytes using the water-soluble tetrazolium salt-1 (WST-1) assay. GUE (≤50 µg/mL) and LicoD (≤10 µM) did not reduce the viability of AML12 (Figure 1C,D).

### 2.2. Regulation of Glucose Metabolism by GUE or LicoD in IR Hepatocytes

**Figure 2:**
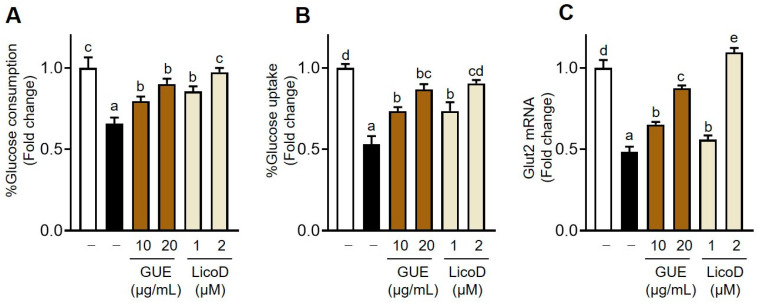
GUE and licochalcone D (LicoD) improved the glucose metabolism in insulin-resistant hepatocytes. AML12 cells were cultured in Dulbecco’s Modified Eagle Medium/F12 containing 2% fetal bovine serum (FBS) without insulin for 24 h. Cells were then exposed to 27 mM glucose and insulin (1 nM) for 24 h with or without GUE (10 or 20 µg/mL) or LicoD (1 or 2 µM). (**A**) Glucose consumption, (**B**) glucose uptake, and (**C**) *Glut2* mRNA expression were determined. Data are expressed as mean ± standard deviation (*n* ≥ 3). The different letters indicate significant differences (*p* < 0.05) as determined by one-way ANOVA followed by Tukey’s post hoc test. ns, not significant.

Exposure of hepatocytes to 27 mM of glucose and insulin for 24 h significantly decreased glucose consumption and uptake, as well as *Glut2* mRNA expression, thereby successfully establishing the IR model of AML12 cells (Figure 2A–C), consistent with previous reports [22]. Treatment with GUE or LicoD dose-dependently increased glucose consumption and uptake in high-glucose-induced IR AML12 cells (Figure 2A,B). Furthermore, GUE (10 or 20 µg/mL) or LicoD (2 µM) mitigated the reduction in *Glut2* mRNA expression under IR conditions (Figure 2C). These results demonstrate that GUE or LicoD effectively alleviated abnormal glucose disposal in hepatocytes caused by high glucose.

### 2.3. Enhancement of the IRS/PI3K Pathway by GUE or LicoD in IR Hepatocytes

**Figure 3:**
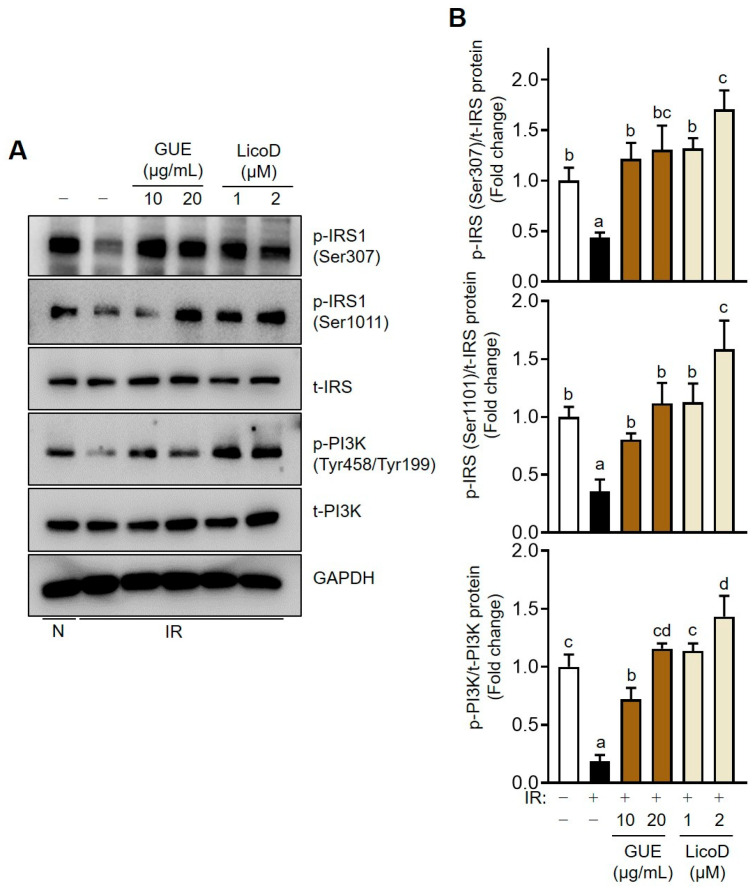
GUE and licochalcone D (LicoD) upregulated the IRS/PI3K signaling pathway in insulin-resistant hepatocytes. AML12 cells were cultured in Dulbecco’s Modified Eagle Medium/F12 containing 2% FBS without insulin for 24 h. Cells were exposed to 27 mM glucose and insulin (1 nM) for 24 h with or without GUE (10 or 20 µg/mL) or LicoD (1 or 2 µM). (**A**) Expression levels of p-IRS1 (Ser307), p-IRS1 (Ser1011), t-IRS1, p-PI3K (Tyr458/Tyr199), t-PI3K, and GAPDH were analyzed using Western blot assay. (**B**) The quantitative expressions of p-IRS1 (Ser307)/t-IRS1, p-IRS1 (Ser1011)/t-IRS1, and p-PI3K (Tyr458/Tyr199)/t-PI3K are shown as bar graphs. Data are expressed as mean ± standard deviation (*n* ≥ 3). The different letters indicate significant differences (*p* < 0.05) as determined by one-way ANOVA followed by Tukey’s post hoc test. ns, not significant.

To further elucidate the protective role of GUE and LicoD against high-glucose-induced abnormalities in glucose metabolism, we analyzed the expression levels of key insulin signaling proteins. Consistent with the patterns observed in glucose consumption and uptake, the phosphorylation levels of IRSs (Ser 307 or Ser 1011) and PI3K (Tyr458/Tyr199) significantly decreased in high-glucose-induced IR AML12 cells compared with normal cells (Figure 3). Interestingly, GUE or LicoD treatment dose-dependently increased phosphorylation in the IRS/PI3K pathway, indicating an association between the activation of IRS/PI3K signaling by GUE or LicoD and their therapeutic effect on the high-glucose-induced downregulation of glucose metabolism (Figure 3).

### 2.4. Inhibition of Akt-Mediated Gluconeogenesis by GUE or LicoD in IR Hepatocytes

**Figure 4:**
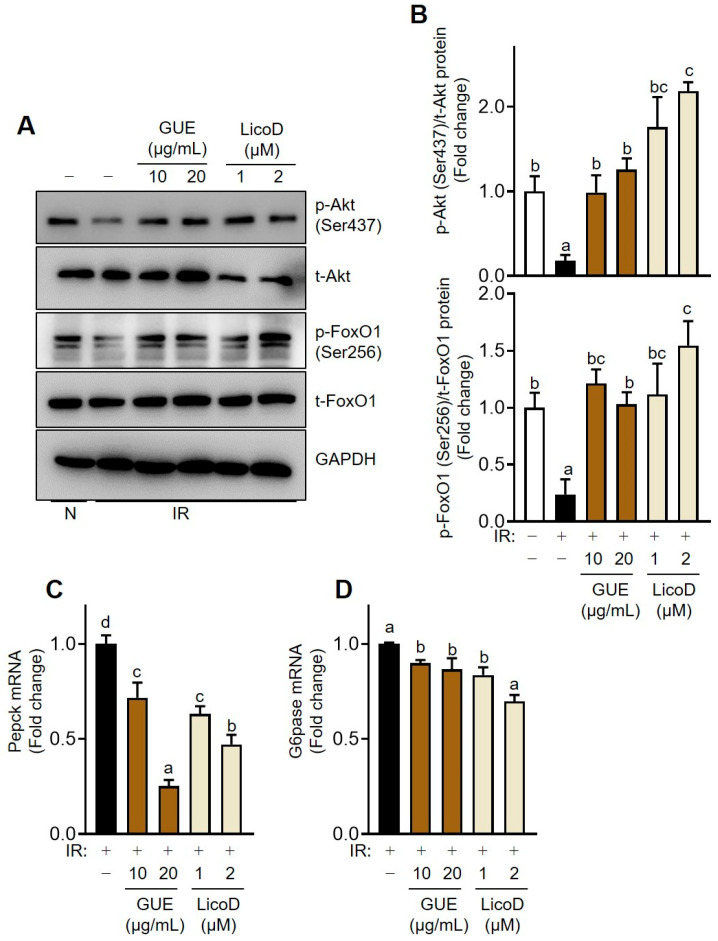
GUE and licochalcone D (LicoD) inhibited Akt-mediated gluconeogenesis in insulin-resistant hepatocytes. AML12 cells were cultured in Dulbecco’s Modified Eagle Medium/F12 containing 2% FBS without insulin for 24 h. Cells were exposed to 27 mM glucose and insulin (1 nM) for 24 h with or without GUE (10 or 20 µg/mL) or LicoD (1 or 2 µM). (**A**) Expression levels of p-Akt (Ser437), t-Akt, p-FoxO1 (Ser256), t-FoxO1, and GAPDH were analyzed using Western blot assay. (**B**) The quantitative expressions of p-Akt (Ser437)/t-Akt and p-FoxO1 (Ser256)/t-FoxO1 are presented as bar graphs. mRNA expression of (**C**) *Pepck* and (**D**) *G6pase*. Data are expressed as mean ± standard deviation (*n* ≥ 3). The different letters indicate significant differences (*p* < 0.05) as determined by one-way ANOVA followed by Tukey’s post hoc test. ns, not significant.

Insulin signaling in hepatocytes regulates glycogen synthesis and gluconeogenesis through Akt activation, which is mediated by the IRS/PI3K signaling pathway. We examined the effect of GUE or LicoD on Akt phosphorylation and its downstream targets that are associated with gluconeogenesis (Figure 4). p-Akt (Ser437) levels decreased in high-glucose-induced IR AML12 cells. Consistent with the upstream IRS/PI3K pathway, p-Akt expression was significantly downregulated following GUE or LicoD treatment in high-glucose-induced IR AML12 cells (Figure 4A,B). Furthermore, p-FoxO1 (Ser256) levels decreased in high-glucose-induced IR AML12 cells, indicating the upregulation of gluconeogenesis, even under glucose-induced conditions (Figure 4A,B). However, GUE or LicoD treatment significantly increased p-FoxO1 levels and decreased *Pepck* and *G6pase* mRNA levels (Figure 4C,D). These results indicate that GUE or LicoD alleviated hepatic IR by suppressing the expression of genes associated with hepatic glucose production.

### 2.5. Enhancement of Glycogen Synthesis by GUE or LicoD in IR Hepatocytes

**Figure 5:**
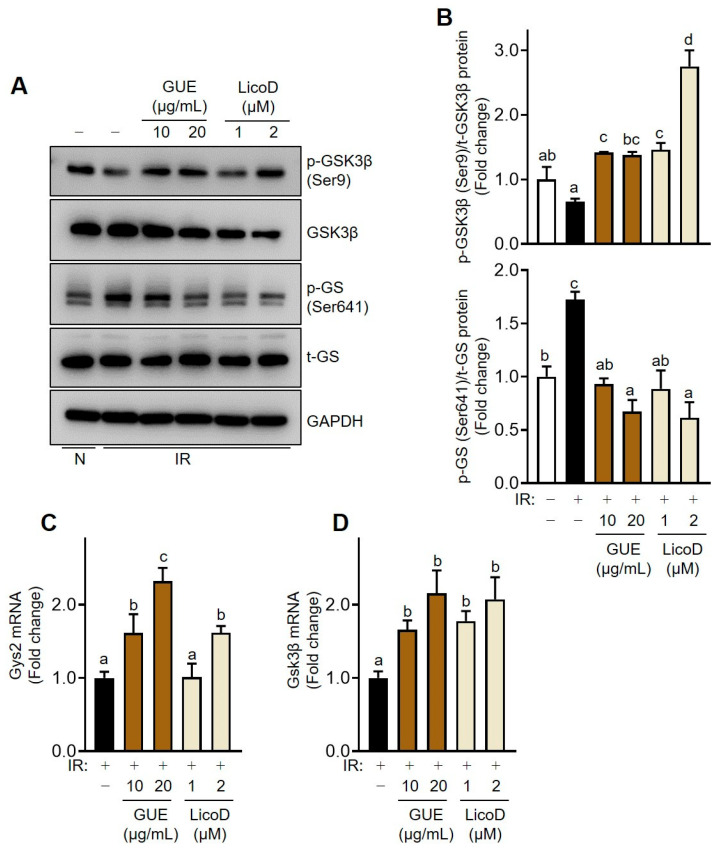
GUE and licochalcone D (LicoD) enhanced glycogen synthesis in insulin-resistant hepatocytes. AML12 cells were cultured in Dulbecco’s Modified Eagle Medium/F12 containing 2% FBS without insulin for 24 h. Cells were exposed to 27 mM glucose and insulin (1 nM) for 24 h with or without GUE (10 or 20 µg/mL) or LicoD (1 or 2 µM). (**A**) Expression levels of p-GSK3β (Ser9), t-GSK3β, p-GS (Ser641), t-GS, and GAPDH were analyzed using Western blot assay. (**B**) The quantitative expressions of p-GSK3β (Ser9)/t-GSK3β (Ser641) and p-GS (Ser256)/t-GS are presented as bar graphs. mRNA expression of (**C**) *Gys2* and (**D**) *Gsk3β*. Data are expressed as mean ± standard deviation (*n* ≥ 3). The different letters indicate significant differences (*p* < 0.05) as determined by one-way ANOVA followed by Tukey’s post hoc test. ns, not significant.

To further explore the effects of GUE or LicoD on glycogen synthesis, we examined the levels of p-Gsk3β (Ser9) and p-GS (Ser641), key molecules involved in the regulation of glycogen synthase via insulin signaling (Figure 5). High glucose treatment inhibited glycogen synthesis, as evidenced by the upregulation of p-GS (Ser641) expression in treated cells (Figure 5A,B). Notably, GUE or LicoD treatment downregulated the high-glucose-induced increase in p-GS (Ser641) levels and dose-dependently increased p-Gsk3β (Ser9) expression (Figure 5A,B). Furthermore, qRT-PCR analysis revealed that *Gys2* and *Gsk3β* mRNA expression was significantly upregulated by GUE or LicoD treatment in high-glucose-induced IR AML12 cells (Figure 5C,D). These findings show that GUE and LicoD enhanced glycogen synthesis by activating enzymes involved in glycogen synthesis in IR hepatocytes.

### 2.6. Improvement of the PPARα/PGC1α Pathway by GUE or LicoD in IR Hepatocytes

**Figure 6:**
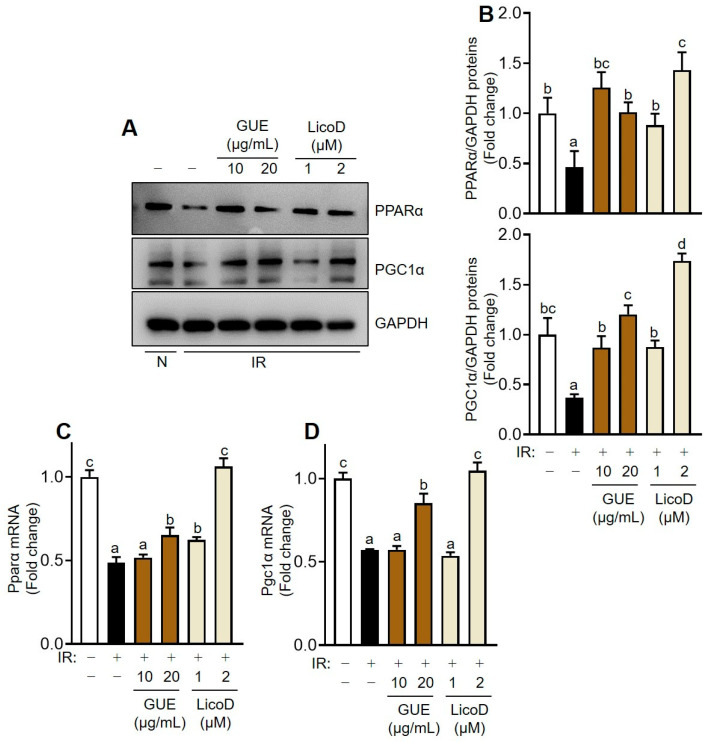
GUE and licochalcone D (LicoD) upregulated the PPARα/PGC1α pathway in insulin-resistant hepatocytes. AML12 cells were cultured in Dulbecco’s Modified Eagle Medium/F12 containing 2% FBS without insulin for 24 h. Cells were exposed to 27 mM glucose and insulin (1 nM) for 24 h with or without GUE (10 or 20 µg/mL) or LicoD (1 or 2 µM). (**A**) Expression levels of PPARα, PGC1α, and β-actin were analyzed using Western blot assay. (**B**) The quantitative expressions of PPARα/β-actin and PGC1α/β-actin are presented as bar graphs. mRNA expression of (**C**) *Pparα* and (**D**) *Pgc1α*. Data are expressed as mean ± standard deviation (*n* ≥ 3). The different letters indicate significant differences (*p* < 0.05) as determined by one-way ANOVA followed by Tukey’s post hoc test. ns, not significant.

**Figure 7:**
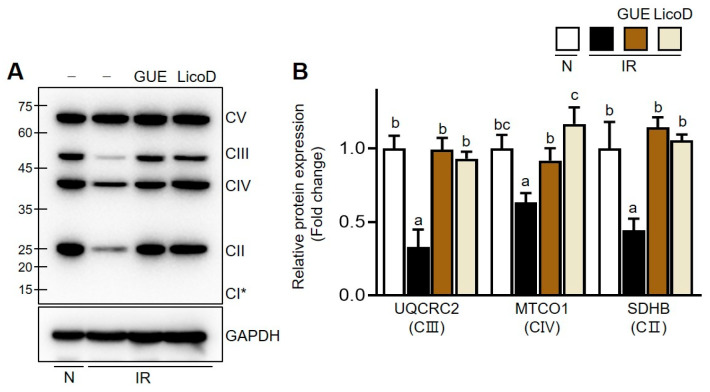
GUE and licochalcone D (LicoD) increased expression of oxidative phosphorylation (OXPHOS) complex proteins in insulin-resistant hepatocytes. AML12 cells were cultured in Dulbecco’s Modified Eagle Medium/F12 containing 2% FBS without insulin for 24 h. Cells were exposed to 27 mM glucose and insulin (1 nM) for 24 h with or without GUE (20 µg/mL) or LicoD (2 µM). (**A**) Subunits of the oxidative phosphorylation (OXPHOS) complex (C) were analyzed using Western blot assay. (**B**) The quantitative expressions of UQCRC2/GAPDH, MTCO1/GAPDH, and SDHB/GAPDH are presented as bar graphs. Data are expressed as mean ± standard deviation (*n* ≥ 3). The different letters indicate significant differences (*p* < 0.05) as determined by one-way ANOVA followed by Tukey’s post hoc test. ns, not significant. CV, ATP synthase subunit alpha (ATP5A); CIII, ubiquinol-cytochrome C reductase core protein 2 (UQCRC2); CIV, mitochondrially encoded cytochrome C oxidase I (MTCO1); CII, succinate dehydrogenase subunit B (SDHB); CI, NADH dehydrogenase beta subcomplex subunit 8 (NDUFB8). The CI subunit (with a theoretical molecular weight of ~20 kD) was not detected.

Finally, we investigated the effects of GUE or LicoD on mitochondrial dysfunction in high-glucose-induced IR hepatocytes. The PPARα/PGC1α signaling pathway is crucial in mitochondrial energy metabolism and is closely linked to mitochondrial activation. Consistent with previous reports [23,24], PPARα and PGC1α expression significantly decreased in IR hepatocytes (Figure 6A,B). However, GUE or LicoD treatment dose-dependently mitigated this suppression of PPARα/PGC1α protein expression, as confirmed by mRNA analysis (Figure 6C,D). These data underscore the importance of PPARα/PGC1α expression, under induction by GUE or LicoD, in restoring mitochondrial function in IR hepatocytes. Furthermore, the mitochondrial DNA content encoding Cyb (mitochondrial complex III) and Co2 (mitochondrial complex IV) decreased in high-glucose-induced IR AML12 cells (Figure S1). In contrast, GUE or LicoD treatment promoted the increase in mitochondrial DNA mass in these cells (Figure S1). Furthermore, mitochondrial OXPHOS proteins (complexes II, III, and IV) were significantly downregulated in high-glucose-induced IR AML12 cells, which was reversed by treatment with GUE or LicoD (Figure 7 and Figure S2). Thus, our in vitro data show that GUE and LicoD effectively improved glucose metabolism in IR hepatocytes by activating PPARα/PGC1α signaling, thereby promoting mitochondrial biogenesis and protein quality control.

## 3. Discussion

Few studies have investigated the effects of LicoD, a chalcone compound derived from licorice, on metabolic diseases, such as obesity and NAFLD, compared with other licochalcone derivatives, such as LicoA and LicoB. Previous studies have shown that LicoA inhibits fat accumulation and enhances FA transport in hepatocytes while promoting FA oxidation in differentiated adipocytes and lowering blood glucose levels in a high-fat diet (HFD)-induced obesity mouse model [25]. LicoB alleviates drug-induced hepatotoxicity in the mouse liver [26]. Furthermore, crude licorice extract, along with LicoA and LicoB, can enhance cell proliferation in aged C2C12 cells, indicating potential antiaging properties [27]. Although *G. uralensis* has been recognized for its various benefits against metabolic disorders, such as T2DM and obesity, the mechanisms by which its components improve IR-induced metabolic abnormalities have remained unclear [28,29]. In the present study, we investigated whether GUE and LicoD could ameliorate dysregulated glucose metabolism in IR hepatocytes and found that both enhanced glucose availability in high-glucose-induced IR AML12 cells by activating the IRS/PI3K/Akt signaling pathway, thereby promoting glycogen synthesis. Our results demonstrate the potential of these two products as natural therapeutic agents for alleviating IR-mediated metabolic diseases.

Following food intake, pancreatic β-cells detect elevated blood glucose levels and secrete insulin to promote anabolic processes in glucose-consuming organs, including the liver, adipose tissue, and skeletal muscle [30]. Anabolic activation increases glucose uptake from the bloodstream and promotes glucose utilization and storage [31]. Furthermore, insulin inhibits hepatic glucose production by suppressing the expression of gluconeogenic genes, such as *Pepck* and *G6pase*, thereby preventing hyperglycemia [32]. The intracellular actions of insulin begin with the activation of insulin receptor tyrosine kinase, which triggers the phosphorylation of IRS, which subsequently activates downstream targets, including the PI3K/Akt pathway, to maintain glucose homeostasis [33]. Recently, supplementation with natural compounds has been reported to prevent and improve IR-induced hyperglycemia. For example, ginsenoside Rg5, a major ginseng saponin, has been shown to improve hepatic glucose metabolism by activating the IRS/PI3K/Akt insulin signaling pathway, thereby resulting in increased glucose uptake and glycogen synthesis [34]. Cyanidin-3-*O*-glucoside was found to reverse IR and enhance glucose metabolism in IR hepatocytes and a diabetic mouse model by upregulating IRS phosphorylation [35]. In the present study, we found that the IRS-mediated PI3K pathway remained impaired even during glucose–insulin stimulation in IR hepatocytes. However, treatment with GUE or LicoD reactivated the downregulated phosphorylation of IRS/PI3K and suppressed the expression of gluconeogenesis-related genes in IR hepatocytes by modulating their transcriptional regulator, FoxO1. Furthermore, PI3K phosphorylates Akt and inhibits GSK3, thereby promoting glycogen synthase activity and glycogen synthesis. These findings indicate that GUE and LicoD can reduce blood glucose levels by promoting glucose storage and inhibiting glucose production in the hepatic IR state, thereby preventing hyperglycemia-induced metabolic diseases.

PPARα is a nuclear receptor that is critical in regulating lipid and glucose homeostasis [36]. Notably, PPARα contributes to the progression of IR-induced metabolic diseases, including T2DM [37]. PPARα activation enhances glucose uptake and utilization, particularly in the context of IR [38]. The significant role of PPARα in glucose metabolism has been the focus of pharmaceutical research on developing PPARα agonists as potential therapeutic agents for T2DM [39]. In hepatocytes, PPARα is essential for mitochondrial function, as it promotes mitochondrial biogenesis through its coactivator, PGC-1α [40]. Since mitochondria are central to cellular energy production and their dysfunction is influential to the development of metabolic disorders, including T2DM, impaired mitochondrial protein integrity can thus exacerbate IR and disrupt glucose homeostasis [41]. Therefore, activating the PPARα/PGC1α pathway can potentially mitigate IR and manage IR-induced metabolic diseases by enhancing mitochondrial quality [42]. We found that PPARα/PGC1α expression was significantly reduced in IR hepatocytes compared with normal ones. However, treatment with GUE or LicoD restored the expression of proteins and mRNAs in the PPARα/PGC1α pathway. Furthermore, the mitochondrial DNA content significantly increased in IR hepatocytes treated with GUE and LicoD. Unsurprisingly, since mitochondrial DNA encodes proteins essential for mitochondrial function, mitochondrial OXPHOS proteins were upregulated by GUE and LicoD treatment. These results indicate that GUE and LicoD enhance mitochondrial oxidative function under IR conditions by improving mitochondrial integrity, thereby helping mitigate IR-induced metabolic disorders.

The root of *G. uralensis*, one of the oldest medicinal plants, has been historically used to alleviate heat, cough, and pain [43]. Its pharmacological effects and molecular mechanisms of action have been extensively studied. For example, the MeCl_2_ fraction of *G. uralensis* suppressed lipid accumulation and induced adipocyte browning in 3T3-L1 adipocytes, as demonstrated in an HFD-induced obesity mouse model [25]. *G. uralensis* root also exhibited significant hepatoprotective effects against alcohol-induced liver injury [44]. Furthermore, *G. uralensis* extract showed no hepatoxicity and reduced HFD-induced weight gain and blood glucose and insulin levels in mice, likely due to the suppression of de novo lipogenesis-related genes, including sterol regulatory element-binding protein 1 (*Srebp1*), FA synthase (*Fas*), and acetyl-CoA carboxylase (*Acc*) [45]. A recent study on a T2DM mouse model showed that *G. uralensis* root effectively suppressed high-glucose-induced TGF-β1 production by regulating Smad/Stat3-mediated mechanisms, potentially attenuating fibrosis-associated diabetic nephropathy [46]. In the present study, 20 µg/mL of GUE and 2 µM of LicoD exhibited comparable effects in improving glucose metabolism in IR hepatocytes. Our quantitative LC-MS data indicated that *G. uralensis* extract contains 8.61 µg/100 mg of dry weight LicoD. Therefore, the efficacy of *G. uralensis* extract in alleviating metabolic diseases may be partially attributed to LicoD. Further studies are necessary to evaluate the therapeutic potential of LicoD in treating metabolic diseases, such as NAFLD, T2DM, and obesity. Furthermore, the current findings should be validated using both in vivo and in vitro models.

## 4. Materials and Methods

### 4.1. Materials and Reagents

LicoD (purity: >99.8%) was purchased from MedChem Express (#HY−N4187; Monmouth Junction, NJ, USA). Mouse liver AML12 cell lines were obtained from American Type Culture Collection (Manassas, VA, USA). Glucose detection kits were obtained from Abcam (#ab136955; Cambridge, MA, USA). 2-(N-(7-nitrobenz-2-oxa-1,3-diazol-4-yl)amino)-2-deoxyglucose (2-NBDG, #N13195), dimethyl sulfoxide (#276855), D-(+)-glucose solution (100 g/L, #G8644), insulin (#I6634), and dexamethasone (#D1756) were purchased from Sigma-Aldrich (St. Louis, MO, USA). The primary antibodies used were p-IRS-1 (Ser307, #2381; Cell Signaling, Danvers, MA, USA); p-IRS-1 (Ser 1101, Cell Signaling); p-PI3K (Tyr458/Tyr199, #17366; Cell Signaling); PI3K (#4255; Cell Signaling); p-FoxO1 (Ser473, #4255, Cell Signaling); FoxO1 (#2880, Cell Signaling); p-Akt1 (Ser473, #05-736; Sigma-Aldrich); Akt1 (#sc-5298; Santa Cruz Biotechnology, Paso Robles, CA, USA); p-GSK-3β (Ser9, #5558; Cell Signaling); GSK-3β (#9315; Cell Signaling), p-GS, Ser641, #3891; Cell Signaling); GS (#sc-81173, Santa Cruz Biotechnology); peroxisome proliferator-activated receptor α (PPARα, #sc-9000; Santa Cruz Biotechnology); β-actin (#sc-47778; Santa Cruz Biotechnology); total OXPHOS cocktail (#ab110413; Abcam); GAPDH (#2118; Cell Signaling); and α/β-tubulin (#2148; Cell Signaling). Solvents used for LC-MS analysis were purchased from Fisher Scientific Korea Ltd. (Seoul, Republic of Korea).

### 4.2. Extraction and Identification of LicoD from G. uralensis

*G. uralensis* was cultivated in Yeongwol-gun (Gangwon province, Republic of Korea) and harvested in November 2017. *G. uralensis* extract was prepared as described previously [47]. Briefly, *G. uralensis* root was washed and dried. To prepare GUE, the dried sample was extracted twice using 70% EtOH (100 g/L) using a reflux cooling extraction method at 50 °C for 3 h. The extract was filtered, concentrated under reduced pressure, and freeze-dried. To prepare GUH, the dried sample was crushed and extracted twice by reflux cooling extraction at 50 °C for 3 h with distilled water 20 times the sample weight. Afterward, the extract was filtered and freeze-dried. The extract was stored at −20 °C until further use.

The freeze-dried samples were extracted with methanol for 3 h at 40 °C by sonication and filtered through a 0.22 µm pore filter syringe for UPLC–MS analysis (Waters, Milford, MA, USA). LicoD was isolated under the following conditions: column (C18, 1.7 µm, 2.1 × 100 mm, Waters); column temperature, 40 °C; and flow rate, 0.3 mL/min. The sample was eluted using a gradient system of H₂O (0.1% formic acid, solvent A) to acetonitrile (0.1% formic acid, solvent B), starting with 2% B and holding for 1.5 min; increasing to 15% B over 1.5 min and holding for 1 min; increasing to 35% B over 4 min and holding for 1 min; increasing to 90% B over 6 min and holding for 1.5 min; and decreasing to 2% B over 2 min and holding for 2 min. The mass spectrometer was set to multiple reaction monitoring mode to monitor the transition of LicoD: *m*/*z* 353.2 [M − H]^−^ → 150.0 (quantifier) and 338.2 (qualifier). The optimal MS settings for quantification of LicoD were as follows: capillary voltage, 3.0 kV; cone voltage, 30 V; source temperature, 150 °C; desolvation temperature, 350 °C; desolvation gas flow rate, 650 L/h; cone gas flow, 150 L/h; and nebulizing gas flow, 7.0 L/min. Calibration curves (y = 104.56x − 134.85; R^2^ = 0.990) were constructed using a standard of LicoD (1–50 µg/L). The LicoD content was quantitatively analyzed in triplicate. Data analysis was performed using MassLynx V4.1 software (Waters).

### 4.3. Cell Culture and Treatments

AML12 cells were cultured in Dulbecco’s Modified Eagle Medium/F-12 (DMEM/F12, Gibco, Carlsbad, CA, USA) supplemented with 10% fetal bovine serum (FBS, Welgene, Gyeongsan, North Gyeongsang province, Republic of Korea), 1% antibiotic/antimycotic solution (Welgene) and 1% insulin–transferrin–selenium solution (10 μg/mL insulin, 5.5 μg/mL transferrin, and 5 ng/mL selenite; Invitrogen, Carlsbad, CA, USA) at 37 °C and 5% CO₂. The cells were sub-cultured before reaching 90% confluence. AML12 cells (3 × 10^5^ cells) were seeded into a 6-well plate and incubated for 24 h. To induce IR, the culture medium was replaced with DMEM/F12 containing 2% FBS without insulin for starvation. After 24 h of incubation, the cells were exposed to 27 mM glucose and insulin (1 nM) for 24 h with or without GUE or LicoD treatment at specified concentrations.

### 4.4. Cell Viability Assay

Cell viability was determined using the WST-1 assay (Roche Applied Science, Mannheim, Germany). After seeding cells into 96-well plates at a density of 2 × 10^4^ cells per well, the cells were incubated with different concentrations of GUE (0–50 μg/mL) or LicoD (0–10 μM). After 24 h of incubation, 10% WST-1 solution was added to the culture medium. The cells were then incubated for an additional 3 h at 37 °C under a 5% CO_2_ atmosphere. The resulting formazan products were measured at 450 nm using a microplate spectrophotometer (Molecular Devices, Sunnyvale, CA, USA).

### 4.5. Glucose Consumption Assay

We used a colorimetric glucose assay kit (#ab136955, Abcam) to determine cellular glucose consumption. Cells were seeded into a 6-well plate and cultured for 24 h. The culture medium was replaced with FBS-free medium for 30 min. 2-Deoxyglucose (2-DG) was added to the cells, and the mixture was incubated for 20 min at 37 °C to metabolize 2-DG into 2-DG-6-phosphate (2-DG6P). After washing with phosphate-buffered saline (PBS), cells were lysed with extraction buffer. Following a heating and cooling treatment, the cells were neutralized, and the cell supernatant was obtained using centrifugation at 10,000× *g* for 5 min. To generate NADPH from the accumulated 2-DG6P, a reaction mixture was added, and absorbance was analyzed at 412 nm at regular intervals for 10 min.

### 4.6. Glucose Uptake Assay

Cells were seeded into a 6-well plate and then exposed to FBS-free conditions for 1 h. Subsequently, the cells were treated with insulin (1 µM) and then with 0.1 mM 2-NBDG and incubated for 40 min. The cells were washed with PBS and collected through trypsinization. The fluorescence intensity of the cell pellet in FBS-free medium was measured using a microplate fluorescent reader (Ex/Em = 490/550 nm; Molecular Devices).

### 4.7. Quantitative Real-Time Polymerase Chain Reaction

Total RNA was extracted from cells using an RNeasy Mini Kit (Qiagen, Hilden, Germany) and used to synthesize cDNA using a cDNA reverse transcription kit (Toyobo Co., Ltd., Osaka, Japan) following the manufacturer’s instructions. Quantitative real-time polymerase chain reaction (qRT-PCR) was performed using a SYBR Green Master Kit (#4913914001; Roche). The reaction was performed on a CFX Connect™ Real-Time PCR Detection System (Bio-Rad, Hercules, CA, USA). The primer sequences for target genes are listed in Table S1. The relative quantification of mRNA was calculated using the 2^−ΔΔCt^ method and normalized to housekeeping genes.

### 4.8. Western Blot Assay

Total proteins were extracted from cells using a radioimmunoprecipitation assay buffer (#R0278; Sigma-Aldrich) containing protease and phosphatase inhibitors (Roche). The protein concentration was determined using a bicinchoninic acid kit (#BCA1; Sigma-Aldrich). Equal amounts of proteins were separated by 8% sodium dodecyl sulfate–polyacrylamide gel electrophoresis and transferred to polyvinylidene fluoride membranes (Bio-Rad). After blocking the membranes with 5% nonfat dry milk in Tris-buffered saline containing Tween-20 (TBST) for 1 h, the membranes were incubated overnight with specific primary antibodies at 4 °C. The membranes were then washed with TBST for 1 h and incubated with the secondary antibody for 1 h at room temperature. Proteins were detected with an enhanced chemiluminescence reagent (Thermo Fisher Scientific, Sunnyvale, CA, USA). Band intensities were quantified using ImageJ (version 1.54d).

### 4.9. Mitochondrial DNA Quantification

DNA was isolated from the cells using a DNA purification kit (Bioneer Corp., Daejeon, Korea). The ratio of mitochondrial DNA (mtDNA) to genomic DNA was determined by qPCR. The RT-qPCR primers used in this study are listed in Table S1. All primer pairs were run in individual reactions. The final mtDNA/nucDNA ratio for each sample was calculated by averaging the ratios obtained from each primer pair. Expression data were analyzed using the 2^−ΔΔCT^ method.

### 4.10. Statistical Analysis

All statistical analyses were performed using Prism 9 (GraphPad Software, La Jolla, CA, USA). Data were expressed as mean ± standard deviation. Statistical differences were analyzed using one-way analysis of variance (ANOVA), followed by Tukey’s post hoc test for multiple comparisons or by unpaired two-tailed Student’s t-test. Statistical significance was considered at *p*-value < 0.05.

## 5. Conclusions

In conclusion, this study demonstrated the therapeutic potential of LicoD, a chalcone compound derived from *G. uralensis* root, in alleviating various metabolic disorders. LicoD and GUE enhanced glucose metabolism by activating the IRS/PI3K/Akt signaling pathway, improving mitochondrial function, and promoting glycogen synthesis in IR hepatocytes. These results indicate that LicoD from *G. uralensis* is a potential natural therapeutic agent for managing IR-induced metabolic diseases. Further research, including in vitro and in vivo studies, is required to fully validate the therapeutic efficacy of LicoD in metabolic disorders.

## Figures and Tables

**Table 1:** Contents of LicoD in *G. uralensis*.

Sample *	Contents ** (µg/100 mg dry wt.)
GUE	8.61 ± 0.51
GUH	0.85 ± 0.06

* GUE, *G. uralensis* ethanol extract; GUH, *G. uralensis* hot water extracts. ** Data are presented as the mean ± standard deviation (SD; *n* ≥ 3).

## Data Availability

The data presented in this study are available upon request from the corresponding author.

## Supplementary Materials

The following supporting information can be downloaded at https://www.mdpi.com/article/10.3390/ijms251810066/s1: Figure S1: *Glycyrrhiza uralensis* ethanol extract (GUE) and licochalcone D (LicoD) increased mitochondrial DNA (mtDNA) content in insulin-resistant hepatocytes; Figure S2: Effects of *GUE* and licochalcone D LicoD) on oxidative phosphorylation (OXPHOS) complex proteins in insulin-resistant hepatocytes.
