# Supplementary material for: Licochalcone D from Glycyrrhiza uralensis Improves High-Glucose-Induced Insulin Resistance in Hepatocytes
Source: Int J Mol Sci. 2024 Sep 19;25(18):10066. doi: 10.3390/ijms251810066 (PMC11432222; doi:10.3390/ijms251810066)
Supplement: Supplementary file 1 [file ijms-25-10066-s001.zip › Supplementary Material_YG Lee_IJMS.pdf]

Supplementary Material

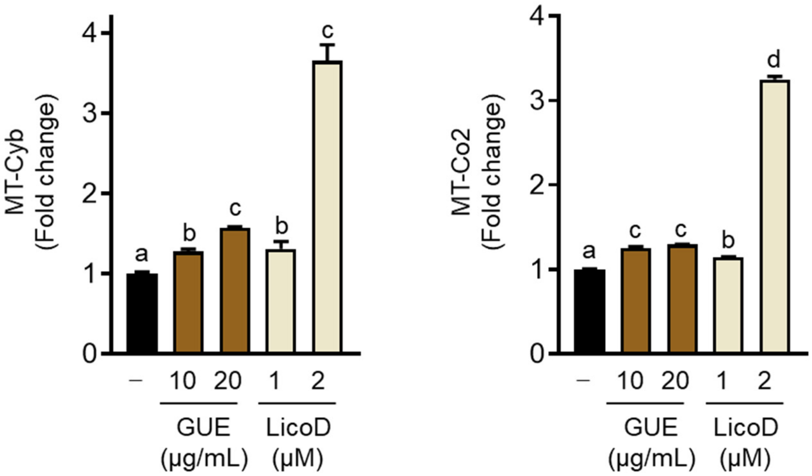

Figure S1. ***Glycyrrhiza uralensis ethanol extract (GUE)*** **and licochalcone D (LicoD) increased mitochondrial DNA (mtDNA) content in insulin-resistant hepatocytes.** AML12 cells were cult ured in Dulbecco's Modified Eagle Medium/F12 containing 2% FBS without insulin for 24 h. Cells were exposed to 27 mM glucose and insulin (1 nM) for 24 h with or without GUE (10 or 20 μg/mL) or LicoD (1 or 2 μM). Relative mtDNA copy number was quantified by RT-qPC R to determine the relative mtDNA content/nuclear DNA. The different letters indicate signific ant differences ($p < 0.05$) as determined by one-way ANOVA followed by Tukey's post hoc te st. ns, not significant. mitochondrially encoded cytochrome B, MT-Cyb; cytochrome C oxidase subunit II, MT-Co2.

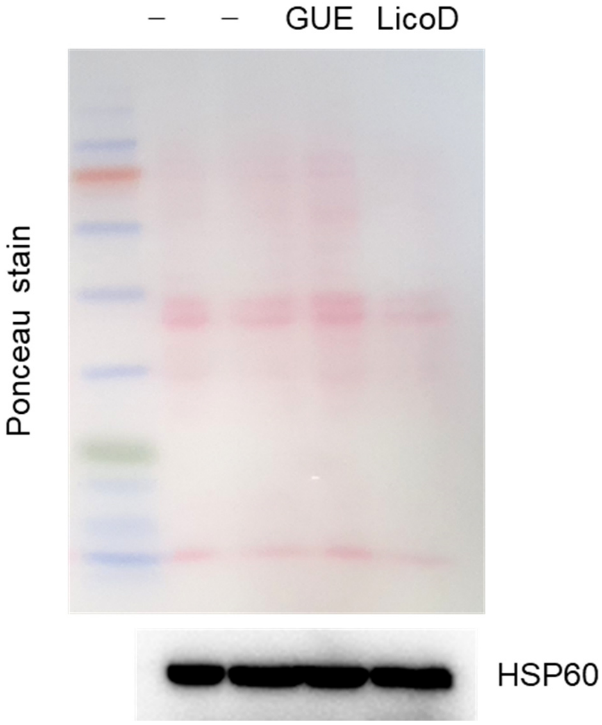

Figure S2. **Effects of GUE and licochalcone D LicoD) on oxidative phosphorylation (OXPHO S) complex proteins in insulin-resistant hepatocytes.** AML12 cells were cultured in Dulbecco's Modified Eagle Medium/F12 containing 2% FBS without insulin for 24 h. Cells were exposed to 27 mM glucose and insulin (1 nM) for 24 h with or without GUE (20 μg/mL) or LicoD (2 μM). The Ponceau stain of the polyvinylidene fluoride membrane is shown to confirm equal p rotein loading. Quantitative analysis of OXPHOS complex proteins was normalized to HSP60 e xpression as the housekeeping gene.
