# Supplementary material for: Licochalcone D from Glycyrrhiza uralensis Improves High-Glucose-Induced Insulin Resistance in Hepatocytes
Source: Int J Mol Sci. 2024 Sep 19;25(18):10066. doi: 10.3390/ijms251810066 (PMC11432222; doi:10.3390/ijms251810066)
Supplement: Supplementary file 1 [file ijms-25-10066-s001.zip › Table S1.pdf]

Table S1. Oligonucleotide sequences used in RT-PCR analysis used in this work.

Target gene	Direction	Sequence (5′-3′)
Glut2	forward	GTGTCTGCTACTGCTCTTCTGTC
	reverse	GACATCCTCAGTTCCTCTTAGTCTC
Pepck	forward	AAGCATTCAACGCCAGGTTC
	reverse	GGGCGAGTCTGTCAGTTCAAT
G6pase	forward	GGACACCGACTACTACAGCAACAG
	reverse	GCATGGCCAGAGGGACTTC
Gys2	forward	TGGGTCTTTAACTGCCTGGTTC
	reverse	TGTTTACCGTCTGCGTGGTC
Gsk3β	forward	GCCGAGTGACAAAGGAAGGA
	reverse	TGGGAAGGAGGGAGGAGATG
Pparα	forward	AGAGCCCCATCTGTCCTCTC
	reverse	ACTGGTAGTCTGCAAAACCAAA
Pgc1α	forward	AATGAGGGCAATCCGTCTTCA
	reverse	AAGTGGTGTAGCGACCAATCG
MT-Cyb	forward	ACGCAAACGGAGCCTCAATA
	reverse	TGTGGCTATGACTGCGAACA
MT-Co2	forward	ACCTGGTGAACTACGACTGC
	reverse	GGACTGCTCATGAGTGGAGG
nDNA H19	forward	GAACAGAAGCATTCTAGGCTGG
	reverse	TTCTAAGTGAATTACGGTGGGTG
Gapdh	forward	CAAGGTCATCCATGACAACTTTG
	reverse	GGCCATCCACAGTCTTCTGG
Rplp0	forward	GTGCTGATGGGCAAGAAC
	reverse	AGGTCCTCCTTGGTGAAC

Note: Glucose Transporter Type 2, *Glut2*; Phosphoenolpyruvate Carboxykinase, *Pepck*; Glucose-6-Phosphatase, *G6pase*; Glycogen Synthase 2, *Gys2*; Glycogen Synthase Kinase 3 beta, *Gsk3β*; Peroxisome Proliferator Activated Receptor Alpha, *Pparα*; Peroxisome proliferator-activated receptor γ coactivator 1 α, Pgc1α; Mitochondrially Encoded Cytochrome B, MT-Cyb; Cytochrome C Oxidase Subunit II, *MT-Co2*; Nuclear DNA H19, nDNA H19; Glyceraldehyde-3-phosphate dehydrogenase, Gapdh; Ribosomal protein lateral stalk subunit P0, Rplp0.
